# Mangiferin alleviates experimental peri-implantitis via suppressing interleukin-6 production and Toll-like receptor 2 signaling pathway

**DOI:** 10.1186/s13018-019-1387-3

**Published:** 2019-10-17

**Authors:** Hao Li, Zhiyong Chen, Xinghua Zhong, Jiaquan Li, Wei Li

**Affiliations:** 1 Department of Prosthodontics, The Affiliated Hospital of Stomatology, Guangxi Medical University, 10 Shuangyong Road, Nanning, 530021 People’s Republic of China; 2 Department of Immunology and Infectious Diseases, The Forsyth Institute, 245 First Street, Cambridge, 02142 USA; 3 Department of Oral Medicine, Infection and Immunity, Harvard University School of Dental Medicine, 188 Longwood Avenue, Boston, 02115 USA; 4 Department of Stomatology, The First Affiliated Hospital of Guangxi Medical University, 6 Shuangyong Road, Nanning, 530021 People’s Republic of China; 5 Medical Scientific Research Center, Guangxi Medical University, 22 Shuangyong Road, Nanning, 530021 People’s Republic of China; 6 State Key Laboratory of Oral Diseases, West China Hospital of Stomatology, Sichuan University, 14 3rd Section S Renmin Road, Chengdu, 610041 People’s Republic of China

**Keywords:** Mangiferin, Peri-implantitis, Interleukin-6, Toll-like receptor 2, Bone loss

## Abstract

**Background:**

TLR2 (Toll-like receptor 2) signaling and its downstream proinflammatory cytokines are considered to be important in the progression of peri-implantitis. A natural medicine, mangiferin has exhibited modulatory effect on TLR2 signaling and anti-inflammatory effects on different diseases. The objective of the present study is to investigate the effect of mangiferin on peri-implantitis and the potential mechanisms by administering this drug to an experimental peri-implantitis mouse model.

**Methods:**

Maxillary left first, second, and third molars of mice were extracted, and dental implants were placed in the region of the maxillary left second molars. Then, peri-implantitis was induced by tying ligatures around implants, and mangiferin was given orally to the mice. After 6-week mangiferin treatment, bone loss around the implants was detected using micro-computerized tomography (micro-CT). Alveolar bone and inflammatory infiltrate in peri-implant tissues were examined using hematoxylin and eosin (H&E) staining. Production of interleukin-6 (IL6), a TLR2 downstream proinflammatory cytokine, in the tissue surrounding implants was measured using quantitative real-time reverse transcription polymerase chain reaction (qRT-PCR) analysis. IL6 protein expression and TLR2 signaling pathway activation in peri-implant tissues were detected using western blot analysis.

**Results:**

Micro-CT demonstrated reduced bone loss in peri-implantitis upon mangiferin administration. Additionally, H&E staining showed more alveolar bone and less inflammatory infiltrate in peri-implant tissues after mangiferin application. Moreover, qRT-PCR analysis demonstrated lower levels of IL6 gene expression, and western blot analysis showed decreased protein expression of IL6 and TLR2, and suppressed phosphorylation of TLR2 downstream nuclear factor-κB, p38 mitogen-activated protein kinase, and c-Jun N-terminal kinase after mangiferin treatment.

**Conclusions:**

These results suggest the suppressive effect of mangiferin on bone damage and inflammatory infiltrate in peri-implantitis. These therapeutic effects may be associated with inhibited IL6 production and reduced TLR2 signaling activation in peri-implant tissues.

## Background

Currently, dental implants have been widely used to rehabilitate lost teeth. With the increasing use of these implants, peri-implantitis rises in incidence and comprises a great clinical challenge. Peri-implantitis is an inflammatory condition of the soft tissues and alveolar bone around dental implants, which can result in bone destruction and consequent implant failure [1].

Although pathogenic mechanisms underlying peri-implantitis still remain unclear, the excessive inflammatory response caused by microbial pathogens adhering to implant surface and their toxins is believed to play an important role in the progression of this disease [2]. Certain key virulent attributes of peri-implantitis pathogens, such as lipopolysaccharide (LPS), can stimulate host cells in gingival and osseous tissues to overexpress proinflammatory cytokines including interleukin-6 (IL6) [3]. As a critical stimulator of alveolar bone resorption, IL6 can intensify local inflammatory process and aggravate peri-implantitis [4]. Toll-like receptors (TLRs) are a family of pattern recognition receptors that recognize microbial components [5]. LPS can interact with TLR2, a principal member of the TLR family, and subsequently activates TLR2 downstream proteins nuclear factor-κB (NFκB), p38 mitogen-activated protein kinase (MAPK), and c-Jun N-terminal kinase (JNK), regulating the production of LPS-induced proinflammatory cytokines, including IL6 [5]. A recent research also demonstrates the crucial role of TLR2 signaling activation in exacerbated bone loss and inflammatory infiltrate in peri-implantitis [6]. Thus, suppressing the activation of TLR2 signaling may become an effective strategy for peri-implantitis therapy.

Current therapies for peri-implantitis mainly focus on bacterial removal and can be divided into mechanical and chemical treatments [7], but they are not free from disadvantages. Mechanical modalities, such as conventional and ultrasonic scaling, may damage implant surface and fail to regulate the inflammatory disorder in the peri-implant tissues. Chemical treatments, such as antibiotics and chlorhexidine, can cause increases in antibiotic resistant bacteria or tissue irritation, if used frequently [8, 9]. Thus, novel strategies with the goal of inflammatory regulation in peri-implantitis treatment have been highlighted during recent years. Certain proinflammatory protein inhibitors, including tumor necrosis factor α antagonists and IL1 receptor antagonists, have shown anti-inflammatory effects in periodontal diseases and may be used in peri-implantitis treatment [10, 11]. However, they can induce antibody formation in the body, leading to decreased drug efficacy [12, 13]. This potential side effect limits their clinical application.

Accumulating evidences have shown inflammation modulatory properties of different substances in natural plants, suggesting their potential in the treatment of inflammatory diseases [14–16]. Mangiferin (2-C-b-D-glucopyranosyl-1,3,6,7-tetrahydroxyxanthone) is a natural xanthone present in significant levels in different parts of a mango fruit [17]. It has tremendous health-related properties, such as antiviral [18], antioxidative [19], and anti-inflammation effects [20], and exhibits little adverse effects [21]. Its oral LD50 value in mice is 400 mg/kg [21], and it shows no cytotoxicity in cell culture in vitro even used at a high concentration of 100 μM [22]. Currently, there is no report on its detrimental effects on dental implant surface, induction of drug resistance, or antibody formation during treatment. These advantages make this xanthone become a promising candidate for developing natural medicine. Previous reports have shown the inflammation regulatory effects of mangiferin under different inflammatory statuses, including colitis, dermatitis, and periodontitis [23–25]. Additionally, in an in vitro periodontal disease environment, mangiferin reduces IL6 expression and alleviates inflammatory response by inhibiting the activation of TLR signaling [26]. These reports suggest the potential therapeutic effect of mangiferin on peri-implantitis.

In the present study, to investigate the effect of mangiferin on peri-implantitis and the potential mechanisms, we established experimental peri-implantitis in mice and examined alveolar bone, inflammatory infiltrate, and the changes of IL6 expression and TLR2 signaling activation in tissues around implants after mangiferin treatment.

## Methods

### Mice

Sixty, 4-week-old male C57BL/6J wildtype mice were purchased from the Laboratory Animal Center, Guangxi Medical University (Nanning, China). All protocols were approved by the Institutional Committee for Animal Use and Care in Guangxi Medical University. After acclimatization for 1 week before the experiments, the mice were randomly divided into normal control (N), vehicle-treated peri-implantitis (P), and mangiferin-treated peri-implantitis (M) groups (20 mice in each group). Mice were fed a soft diet (Laboratory Animal Center, Guangxi Medical University, Nanning, China) ad libitum for the duration of the experiment.

### Induction of peri-implantitis

Maxillary left first, second, and third molars of all mice were extracted under inhalation anesthesia with 3% isoflurane, and the tooth extraction sites were allowed to heal for 8 weeks [27]. Then, smooth surface screw-shaped titanium implants (National Engineering Research Center for Biomaterials, Sichuan University, Chengdu, China) were placed into the healed extraction sockets as previously reported [27]. Briefly, the mice were anesthetized with 3% isoflurane, and implants (one per animal) were inserted in the region of the maxillary left second molars, after gingival flap removal and osteotomy by drilling [27]. The threaded surface of the implants was 1.0 mm in length and 0.5 mm in diameter, and the implants went into alveolar bone approximately 1.0 mm in depth. Subsequently, the implants were allowed to heal for 4 weeks. After implant healing, peri-implantitis was induced by tying 6–0 silk ligatures around each implant immediately apical to the implant head in vehicle-treated peri-implantitis and mangiferin-treated peri-implantitis groups. No ligatures were placed around the implants in normal control group.

### Treatment with mangiferin

Upon ligature placement, mice in the mangiferin-treated peri-implantitis group were orally given mangiferin (Sigma-Aldrich Co., St Louis, MO, USA) once a day for 6 weeks (dose: 50 mg/kg bodyweight). Saline was used as vehicle, and mice in normal control and vehicle-treated peri-implantitis groups were given only saline. All animals received oral administration of mangiferin or saline from the day of ligature placement and received the last administration the day before they were sacrificed by CO_2_ inhalation. No mice died and all implants existed until the end of the study.

### Micro-computerized tomography analysis

Upon sacrifice,10 mouse maxillae in each group were randomly selected and fixed in 4% paraformaldehyde for examination using micro-computerized tomography (micro-CT) (Skyscan, Kontich, Belgium). All samples were oriented to make the head and the shaft of implants perpendicular to each other in sagittal and coronal planes [27]. Then, they were detected at 70 kVp operating voltage and 114 mA current and 6 mm isotropic voxel resolution, with 200 ms exposure time and 5 frames averaged per view [6]. Afterwards, volumetric data were converted to DICOM format and imported in Dolphin software (Chatsworth, CA, USA) to generate reconstructed images [6]. To quantify the alveolar bone loss, a cylinder with a diameter of 1.0 mm and a height of 1.0 mm is defined as volume of interest (VOI) from the top surface of each implant [6]. The bone loss surrounding implants was calculated by total VOI volume (TV) minus total bone volume (BV) in 3D morphometric analysis [6].

### Histological analysis

After micro-CT examination, the paraformaldehyde fixed maxillae were decalcified with 10% ethylenediaminetetraacetic acid solution, embedded in paraffin, and cut into sections in the mesial-distal plane (5 μm thickness) for hematoxylin and eosin (H&E) staining as previously reported [28]. Stained sections were photographed under a light microscope equipped with a Nikon 80i (Nikon Corp., Tokyo, Japan). At an objective × 40 magnification, alveolar bone tissues surrounding implants in 4-unit squares (100 μm × 100 μm) were analyzed according to Lane-Sandhu histological scoring criteria [29] and then averaged to obtain the histological bone score of each mouse. Meanwhile, at an objective × 40 magnification, numbers of inflammatory cells in 4-unit squares (50 μm × 50 μm) of peri-implant connective tissues were counted and then averaged to represent the level of inflammatory infiltrate per animal [6].

### Quantitative real-time reverse transcription polymerase chain reaction (qRT-PCR) analysis

After sacrifice, the other 10 mouse maxillae in each group were collected and stored at − 80 °C. Total RNA of the tissues around each implant (including soft tissues and alveolar bone tissues) was extracted using TRIzol reagent (TaKaRa, Japan). Then, RNA was quantified and reverse-transcribed into cDNA with Prime-Script RT reagent Kit (TaKaRa, Japan), and the resultant cDNA products were amplified using SYBR Green qPCR Master Mix (TaKaRa, Japan) for real-time PCR analysis as previously described [30]. Primer sequences were as follows: IL6: 5′-GCTGGAGTCACAGAAGGAGTGGC-3′ (forward), 5′-GGCATAAC GCACTAGGTTTGCCG-3′ (reverse), and glyceraldehyde 3-phosphate dehydrogenase (GAPDH): 5′-GGTGAAGGTCGGTGTGAACG-3′ (forward), 5′-CTCGCTCCTGGAAGA TGGTG-3′ (reverse).

### Western blot analysis

Total protein in tissues around each implant (including soft tissues and alveolar bone tissues) was obtained from the mouse maxillae stored at − 80 °C using a ReadyPrep Protein Extraction Kit (BioRad Laboratories, Hercules, CA, USA), and inflammatory cytokine IL6 and proteins in TLR2 signaling pathway were detected using western blot analysis [31]. The primary antibodies were mouse monoclonal anti-IL6 (1:500), anti-GAPDH (1:500), and rabbit monoclonal TLR2 (1:800), anti-total NFκB p65 (anti-NFκB p65) (1:800), anti-phospho-NFκB p65 (1:800), anti-total p38 (anti-p38) (1:500), anti-phospho-p38 (anti-pp38) (1:500), anti-total-JNK (anti-JNK) (1:500), and anti-phospho-JNK (anti-pJNK) (1:500). The secondary antibody was horseradish peroxidase-conjugated anti-mouse (1:3000) or anti-rabbit (1:3000). GAPDH protein was chosen as an internal control, while proteins NFκB p65, p38, and JNK were used as internal controls, when the phosphorylation levels of NFκB p65, p38, and JNK were detected. The immunoreactive bands were examined using enhanced chemiluminescence. All antibodies were from Santa Cruz Biotechnology (Santa Cruz, CA, USA), except the rabbit monoclonal primary antibody from Abcam (Cambridge, MA, USA).

### Statistical analysis

All values are expressed as means ± SD. One-way analysis of variance (ANOVA) followed by the Student-Newman-Keuls *q* test was used to assess the differences between groups. Statistical significance was considered at a *P* value < 0.05. Statistical analyses were performed using Statistical Package for the Social Sciences (SPSS) software (Version 23.0, SPSS Inc., Chicago, IL, USA).

## Results

### Bone loss around implants

**Fig. 1:**
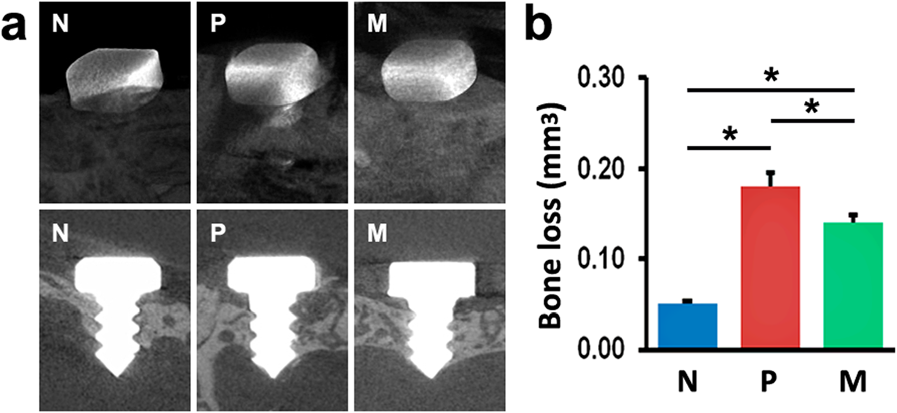
Bone loss around implants of N, P, and M mice was detected using micro-CT at sacrifice. **a** Micro-CT images of alveolar bone surrounding implants. **b** Bone loss was calculated by total VOI volume minus total bone volume. The values are presented as the mean ± SD (*n* = 10, *P < 0.05). N normal control group, P vehicle-treated peri-implantitis group, and M mangiferin-treated peri-implantitis group

Implant survival was 100% in all groups at the end of the experiment. As shown in Fig. 1a, the bone level was more apical in P and M groups, compared with N group, and this level was less apical in M group than in P group. Additionally, micro-CT analysis revealed that the mice in P and M groups exhibited more bone loss around implants, compared with N mice, suggesting the establishment of peri-implantitis models (Fig. 1b). After 6-week mangiferin administration, M mice showed less bone loss compared with P mice (Fig. 1b).

### Histological examination of peri-implant tissues

**Fig. 2:**
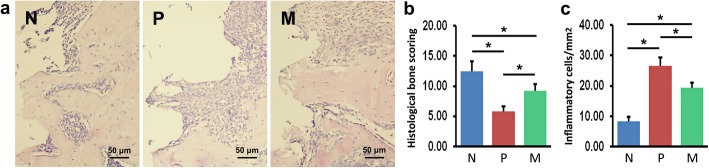
Alveolar bone and inflammatory infiltrate in peri-implant tissues were examined using H&E staining. **a** Images of peri-implant tissues of N, P, and M mice. **b** Histological scoring of alveolar bone surrounding implants of N, P, and M mice. **c** Numbers of inflammatory cells in peri-implant tissues of N, P, and M mice. The values are shown as the mean ± SD (*n* = 10, *P < 0.05). N normal control group, P vehicle-treated peri-implantitis group, and M mangiferin-treated peri-implantitis group

H&E staining showed more alveolar bone tissues surrounding implants in normal control group, compared with both peri-implantitis groups (P and M groups) (Fig. 2a), whereas P group had less bone tissues around implants, compared with M group (Fig. 2a). Lane-Sandhu histological scoring also supported these results (Fig. 2b). Additionally, the results of H&E staining demonstrated greater number of inflammatory cells infiltrated in the connective tissues around implants in both peri-implantitis groups (P and M groups) than in normal control group, suggesting inflammatory response in peri-implant tissues under the peri-implantitis condition (Fig. 2a, c). Moreover, the vehicle-treated peri-implantitis group exhibited the largest amount of inflammatory cell infiltration among the three groups (Fig. 2a, c).

### Effects of mangiferin administration on IL6 production and TLR2 signaling

**Fig. 3:**
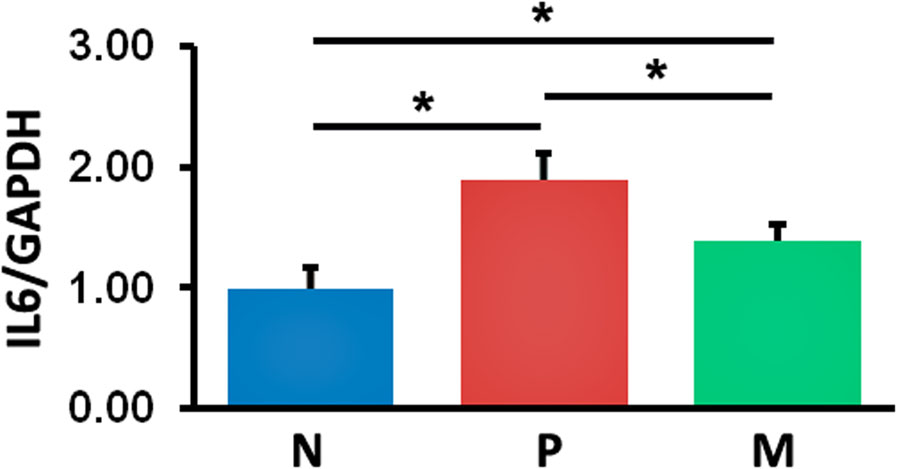
Gene expression levels of IL6 in peri-implant tissues were detected using qRT-PCR analysis. The values presented are the mean ± SD (*n* = 3, *P < 0.05). N normal control group, P vehicle-treated peri-implantitis group, and M mangiferin-treated peri-implantitis group

Based on the results of qRT-PCR, elevated levels of IL6 gene expression were observed in P and M mice, compared with their normal controls; however, these levels were reduced after mangiferin treatment (M mice vs. P mice) (Fig. 3).

**Fig. 4:**
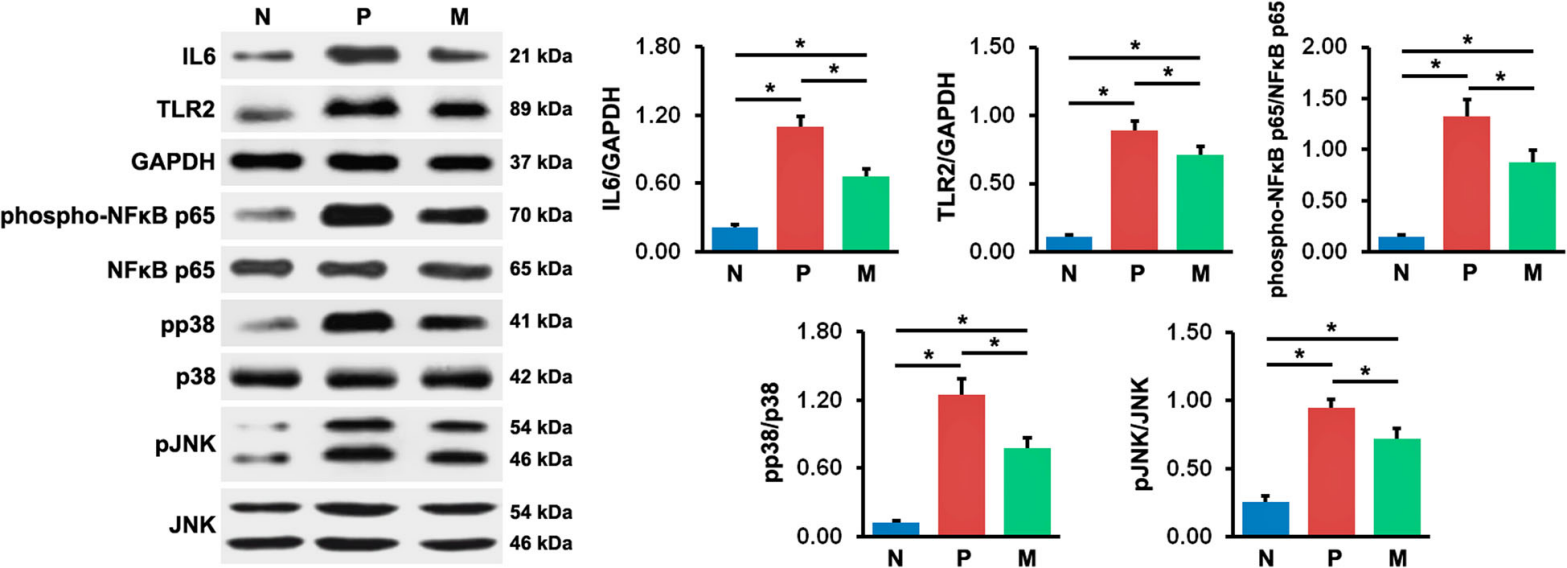
Protein expression levels of IL6 and TLR2, and protein phosphorylation levels of NFκB p65, p38, and JNK in peri-implant tissues were measured using western blot analysis. The values presented are the mean ± SD (*n* = 3, *P < 0.05). N normal control group, P vehicle-treated peri-implantitis group, and M mangiferin-treated peri-implantitis group

The results of western blot analyses were demonstrated in Fig. 4. In agreement with the findings of qRT-PCR, the IL6 protein level in peri-implant tissues in N mice was markedly lower than in P and M mice, and the level was significantly higher in P mice than in M mice. Additionally, the expression of TLR2 protein was greater in all mice with peri-implantitis (P and M mice) than in N mice, whereas it obviously decreased in mice with peri-implantitis upon mangiferin treatment (M mice vs. P mice). Furthermore, the phosphorylation level of NFκB increased in P and M mice, compared with N mice, while it decreased dramatically in M mice, compared with P mice. Consistent with NFκB phosphorylation, p38 phosphorylation was upregulated in P and M mice, compared with the normal controls, and it was downregulated in M mice, compared with P mice. Similarly, the phosphorylation level of JNK was higher in both peri-implantitis groups (M and P groups) than in N group, and in M group than in P group. These findings indicated greater IL6 expression and TLR2 signaling activation in tissues around implants under the condition of peri-implantitis, which might be suppressed by mangiferin administration.

## Discussion

Peri-implantitis is an increasing problem in dentistry, as dental implants are widely used clinically. Recent studies have shown that up to 56% of implant patients and even 43% of implant sites can be ailed by this disease [32]. Peri-implantitis usually exhibits obvious inflammatory response and damage in peri-implant tissues, especially alveolar bone destruction around implants, and subsequently leads to implant loss, if left untreated. In this study, we found more bone loss and less alveolar bone left around implants in all peri-implantitis mice, compared to normal control mice, suggesting the establishment of peri-implantitis. However, these two parameters were improved upon mangiferin application, suggesting the protective effect of mangiferin on peri-implantitis.

Besides bone loss, inflammatory infiltrate in the diseased lesions is considered as another crucial parameter to represent the severity of peri-implantitis [6, 33]. Enhanced density of the infiltrate is correlated with aggravated peri-implantitis [34]. In the current research, we observed greater inflammatory infiltrate in both groups with peri-implantitis, compared with normal control group, demonstrating the inflammation in tissues surrounding implants, whereas this parameter diminished after mangiferin treatment, indicating the anti-peri-implantitis effect of mangiferin.

To further investigate the underlying mechanism of the effect of mangiferin on peri-implantitis, we detected the inflammatory cytokine in peri-implant tissues using qRT-PCR and western blot analyses. Our findings showed that both peri-implantitis groups exhibited greater IL6 production, compared with normal healthy group, while mangiferin-treated peri-implantitis group had less IL6 production, compared with vehicle-treated peri-implantitis group, implying that the therapeutic effect of mangiferin may be associated with reduced IL6 levels. IL6 is a proinflammatory cytokine produced by many cells in response to LPS from peri-implantitis pathogens, and it can promote the proliferation and activation of different immune cells, subsequently stimulating bone resorption potently [35]. Clinical studies have demonstrated the positive correlation between IL6 and peri-implantitis: higher IL6 expression was observed in diseased sites in patients with peri-implantitis, compared to healthy individuals [36]; and IL6 concentration was reduced in peri-implant sulcus fluid in patients with attenuated peri-implantitis and clinically stable treatment outcomes [37].

As reported previously, IL6 is one of the downstream cytokines in TLR2 signaling [38, 39], so we detected the activation of TLR2 pathway in peri-implant tissues using western blot analysis. Our results showed that TLR2 expression levels were in agreement with the severity of peri-implantitis. Upon mangiferin treatment, TLR2 expression decreased in mice with peri-implantitis. These findings implicate that the suppression of peri-implantitis by mangiferin may be correlated with the inhibition of TLR2 expression. Consistent with our results, a recent research shows that TLR2 knockout can alleviate bone loss and inflammatory infiltrate in peri-implantitis mouse models [6]. It has been demonstrated that TLR signaling plays a key role in aggravating inflammatory response in different inflammatory diseases, such as Crohn’s disease and periodontitis [40]. Due to the heterogeneity in lipid A structure, LPS from some pathogens in peri-implantitis can interact with TLR2 and subsequently activate TLR2 downstream pathways, contributing to the production of numerous proinflammatory cytokines, including IL6 [38, 39].

Based on the results of western blot analyses, we found upregulated phosphorylation levels of NFκB, p38 MAPK, and JNK in both peri-implantitis groups, compared with normal group. Upon mangiferin treatment, these phosphorylation levels were significantly downregulated. Our observations suggest that the attenuation of peri-implantitis by mangiferin may be partly a result of the suppressed activation of NFκB, p38 MAPK, and JNK signaling. As shown in previous research, after binding to its cognate pathogen-associated molecular patterns, TLR2 initiates an intracellular signaling cascade through cytoplasmic intermediates, including Myd88, that ultimately results in NFκB and MAPK activation, which enhances the transcription of inflammatory cytokines [41]. NFκB is one of the most principal transcription factors, which plays a crucial role in regulating inflammatory responses [42]. In particular, excessive activation of NFκB p65, a key member of NFκB families, is often found in the inflammation caused by LPS from oral pathogens [43]. In an animal model of experimental periodontitis, enhanced NFκB p65 phosphorylation levels were observed, while the levels were reduced accompanied with ameliorated inflammatory response after mangiferin administration [25]. P38 MAPK and JNK are important members of MAPK superfamily, which are implicated in regulating various inflammatory responses [44, 45]. Excessive phosphorylation of p38 MAPK and JNK has been reported in several inflammatory diseases, such as colitis and osteoarthritis, and the inhibition of the phosphorylation is correlated with the attenuation of inflammatory response [46, 47]. In recent research on a periodontitis-like environment, the overactivation of p38 MAPK and JNK was observed, and mangiferin treatment inhibited their activation and the production of proinflammatory cytokine in a dose-dependent manner [26].

As a natural xanthone, mangiferin has shown suppressive effects on different inflammation-related diseases, such as diabetes [17], Alzheimer’s disease [48], colitis [23], and periodontitis [25]. These inhibitory effects of mangiferin are associated with the repression of multiple inflammatory proteins, including TLR, MAPK, and NFκB families [17, 23, 25, 48]. In this work, we demonstrate the inhibition of TLR2, NFκB p65, p38, and JNK activation in experimental peri-implantitis upon mangiferin treatment, but it still remains to be confirmed whether there are other members in these inflammatory protein families involved. To elucidate the precise mechanism of the suppression of peri-implantitis by mangiferin, further experiments are required, for instance, detecting the expression of different TLR and MAPK members in peri-implant tissues after mangiferin administration and examining the effects of mangiferin on this disease using TLR2 or other TLR gene knockout animal models.

## Conclusions

In conclusion, the present study demonstrates that mangiferin reduces peri-implant bone damage and inflammatory infiltrate in experimental peri-implantitis. The protective effects of mangiferin on this disease may correlate with the inhibition of IL6 production and TLR2 signaling activation. This work extends the previous findings on the modulation of peri-implantitis and may also provide a potential therapeutic strategy against this disease.

## Data Availability

The datasets used and/or analyzed during the present study are available from the corresponding author on reasonable request.

## Abbreviations

BV: Total bone volume
GAPDH: Glyceraldehyde 3-phosphate dehydrogenase
H&E: Hematoxylin and eosin
IL6: Interleukin-6
JNK: c-Jun N-terminal kinase
LPS: Lipopolysaccharide
M: Mangiferin-treated peri-implantitis
MAPK: Mitogen-activated protein kinase
micro-CT: Micro-computerized tomography
N: Normal control
NFκB: Nuclear factor-κB
P: Vehicle-treated peri-implantitis
pJNK: Phospho-JNK
pp38: Phospho-p38
qRT-PCR: Quantitative real-time reverse transcription polymerase chain reaction
SPSS: Statistical Package for the Social Sciences
TLR2: Toll-like receptor 2
TV: Total VOI volume
VOI: Volume of interest
